# Characterisation of Scale Deposits in Drinking Water Pipes by FTIR and ICP-OES

**DOI:** 10.3390/ma19061223

**Published:** 2026-03-20

**Authors:** Paweł Wiercik, Justyna Stańczyk, Justyna Możejko

**Affiliations:** 1 Institute of Environmental Engineering, The Faculty of Environmental Engineering and Geodesy, Wrocław University of Environmental and Life Sciences, Grunwaldzki Square 24, 50-363 Wrocław, Poland; justyna.stanczyk@upwr.edu.pl; 2 ENTAL Instalacje Sp. Z o.o., 18 Kolejowa St., 47-400 Racibórz, Poland

**Keywords:** scale deposits, corrosion, water supply network, biofilm, FTIR spectroscopy, ICP-OES spectroscopy

## Abstract

Attenuated Total Reflection–Fourier Transform Infrared (ATR-FTIR) spectroscopy and Inductively Coupled Plasma–Optical Emission Spectrometry (ICP-OES) are widely used to investigate the chemical structure and elemental composition of materials. However, the combined application of both methods to examine scale deposits in the water supply network has not yet been explored. In this study, scale deposits collected from the inlets of six pipes (steel, cast iron, lead, wooden) were analysed using both techniques. The application of ATR-FTIR and ICP-OES enabled the identification of mineral phases, organics, and structural differences between individual scale layers. Iron oxyhydroxides, together with silica and aluminosilicates, dominated most samples, whereas shower faucet deposit was primarily composed of carbonates and stearates. The combined analytical approach helped to avoid misinterpretation of FTIR data: although the spectrum of lead pipe deposit resembled hydrated lead carbonates, ICP-OES revealed only trace amounts of lead. Differences in crystallinity between successive layers allowed the reconstruction of the deposition process within the pipes. Poorly crystalline iron oxyhydroxides and silica occurred near pipe walls, while more crystalline phases developed closer to the water interface. These results demonstrate that combining ATR-FTIR and ICP-OES provides a reliable framework for interpreting scale deposit composition and formation in water distribution systems.

## 1. Introduction

Drinking water supply utilities are obligated to provide high-quality water throughout their water supply system, including at the consumer’s tap [1]. However, the water supply network cannot be considered as an inert system but rather as a reactor that interacts with the internal aqueous environment. Component leaching, pipe corrosion or microbial growth on the inner pipe surface result from such interactions. Consequently, unwanted deposits are formed, which negatively affect the physical, chemical and biological stability of the water [2,3]. Among the factors that have the greatest influence on scale formation are: pipe material and its age, hydraulic conditions, electrochemistry, and the chemical composition and microbiology of water [1].

Research conducted by Li et al. (2018) [4] showed that deposits formed in various types of pipes differed in thickness, porosity, and mineral composition. Deposits from old iron pipes consist mainly of iron, carbon and oxygen, as well as significant levels of different metals (Ca, Mg, Mn, S, Si, etc.), including harmful heavy metals (Pb, Zn, Cd, Cr, etc.), occurring mainly in the form of oxides, hydroxides, and carbonates (α-FeOOH, Fe_3_O_4_, FeCO_3_, CaCO_3_, SiO_2_, ZnO, etc.) [4,5,6,7,8]. Deposits in drinking water pipes also contain organic matter (e.g., humic substances and proteins), which can additionally form complex compounds with inorganic components [1,9]. Biofilms can also grow on the inner surfaces of pipes walls. Their growth is promoted by adequate nutrient content (the presence of organic matter) and by protection from disinfection [10]. Biofilms may contain pathogens (e.g., *Pseudomonas*, *Mycobacter*, *Klebsiella*, *Helicobacter pylori*), protozoa (e.g., *Cryptosporidium parvum*), manganese-oxidising bacteria, yeast, fungi, algae, and even faecal bacteria (*Escherichia coli*) [7,11,12,13,14].

Corrosion scales can adversely affect water quality in drinking water pipes through the release of harmful components (As, Pb, Cu, Cd, Cr, etc.), increased chlorine demand (resulting from a significant reduction in its effectiveness), and the promotion of growth and release of pathogenic organisms [2,5,15,16].

Moreover, deposits accumulated on the inner surfaces of pipe walls also affect the hydraulic conditions in the network. They reduce hydraulic capacity (lower flow rate at the same pressure), increase linear and local head losses (due to increased wall roughness, reduced flow area and altered geometry at fittings), and contribute to lower pressure at consumer taps [17,18,19].

The release of metals from deposits results from changes in ion concentrations, pH, alkalinity, organic matter, and the presence of iron-oxidising or sulphate-reducing bacteria [1,6,20,21,22]. A decrease in pH increases acidity, which stimulates metal dissolution and causes its release from scale deposits [23,24]. Research by Zhang et al. (2021) showed that greater amounts of manganese were released from corrosion scales under conditions of lower pH, lower alkalinity, higher temperature, and higher sulphate concentrations [25]. The study by Kurajica et al. (2023) revealed that the addition of humic substances resulted in greater release of As and Pb from pipe deposits [1]. Li et al., 2010 [26] found that the presence of iron-oxidising bacteria in water could facilitate the precipitation of iron oxides by converting ferrous to ferric iron, thus contributing to the formation of red water. Metals and pathogens released from deposits into drinking water can lead to various disorders and health problems, including organ damage, diseases, and infections. Excessive concentrations of components that are normally harmless or desirable may also cause adverse effects, such as gastrointestinal disturbances (e.g., Fe, Cu, Mg), organ damage (Fe), developmental disorders in children, neurotoxic effects and impaired concentration (Mn) [14,27,28]. Excessive levels of heavy metals can cause organ dysfunction (Zn, Ni) and organ damage (Pb, Cd, As, Cr), neurological or neurodevelopmental effects (Al, Pb), and may also have carcinogenic effects (Ni, Cr, Cd, As) [3,27,29]. Therefore, to ensure the safety of the water network system, pipe scale deposits should be systematically examined.

Generally, the composition of scale deposits in water networks is examined using different methods: SEM (Scanning Electron Microscopy), EDS (Energy Dispersive X-ray Spectroscopy), XRD (X-ray Diffraction), XPS (X-ray Photoelectron Spectroscopy), XRF (X-ray Fluorescence), ICP, FTIR and sequential extraction methods [4,6,7,30,31]. One of the most effective methods for determining the quantitative elemental composition of the sample, including the simultaneous analysis of multiple metals, is ICP-OES. However, because the samples must be dissolved prior to analysis, information on chemical speciation and mineral structure is lost. In contrast, FTIR provides information on chemical structure, including functional groups and bonds, and thus on qualitative composition. For this reason, FTIR serves as valuable complement to ICP-OES. FTIR is a quick, simple and cost-effective method for identifying chemical compounds and can be used as an indication of organic material formation in the sample and, therefore, of biofilm presence [2]. However, FTIR does not provide information on quantitative composition.

There is limited research in the literature concerning the analysis of scale deposits in water distribution system using FTIR [2,5,32] or ICP [4,5,20,25,33]. To the authors’ knowledge, there are no scientific publications in which FTIR and ICP-OES were used together for this type of research; however, there are papers in which both methods were applied jointly to study galvanic sludges [34] or soil composition [35,36].

In this study, an attempt was made to determine the composition of scale deposits formed inside different water supply pipes made of various materials, including rarely examined wooden pipes. FTIR spectroscopy was employed for the identification of organic constituents, while ICP-OES was used for quantitative multi-element analysis. The deposits were analysed using the Nicolet iN10 integrated infrared microscope with the Nicolet iZ10 external FTIR module and the Thermo Scientific iCAP 7000 Series ICP-OES spectrometer.

To the authors’ knowledge, the combined use of FTIR spectroscopy and ICP-OES to investigate the composition of scale deposits is a novel approach to this issue. The application of ICP-OES enabled the detection of several trace and rare elements such as gallium, boron, barium, and strontium, which have rarely been reported in previous studies of pipe scale deposits. This knowledge contributes to a better understanding of the occurrence of harmful compounds in deposits, which, under unfavourable conditions, could be released into drinking water and reach consumers. Overall, our findings fill an important knowledge gap and may have practical implications for corrosion control, deposit characterisation, and the long-term management of drinking water distribution systems.

## 2. Materials and Methods

### 2.1. Research Area

Most of the sediment-filled water pipe sections were obtained from the Świdnica (Lower Silesian Voivodeship, Poland) water supply network. The city of Świdnica covers an area of more than 21 km^2^ and serves a total population of around 57,000 in the city itself and an additional 10,000 in the surrounding rural areas. Because the analysed pipes can be considered historical and have remained in operation for a very long period, the exact dates of their installation are unknown due to the lack of detailed historical documentation. Nevertheless, in cooperation with the company’s employees, the approximate age of the pipes was estimated. The pipes were handed over to the authors after being taken out of service and brought to the surface during the modernisation of the water distribution system. These pipes originated mainly from the city centre and, with the exception of the lead connection pipe, formed part of the external distribution network.

Wooden water pipes, made from hollowed tree trunks, were the basic material used in the water supply network in Świdnica from the commissioning of the first pipeline in 1601. They were used in both pressure and gravity systems, including the Witoszów Water Supply System built in 1720–1721. Despite their low durability and frequent leaks, wooden pipes were only partially replaced in the 17th and 18th centuries. As late as 1849, two wooden pipelines (upper and lower) supplying water from the Witoszów Hills were still in operation. Ultimately, wooden water pipes were withdrawn from service only in 1934. Lead pipes appear in historical sources from Świdnica in the context of modernisation plans in 1725 and 1734. During this period, lead pipes were purchased with the intention of replacing wooden sections of the Witoszów water supply system.

The first documented use of cast iron water pipes in Świdnica dates back to 1855. Additional sections of cast iron pipelines were laid between 1862 and 1865, replacing the most unreliable parts of the wooden system. The introduction of cast iron with the commissioning of the new waterworks in 1876 was of decisive importance, as this material enabled water to be transported at higher pressures and over longer distances. By the end of the 19th century, cast iron had become the basic material for the municipal water supply network.

Steel began to appear in Świdnica’s water supply infrastructure at the end of the 19th century, mainly in components of reservoirs and water towers. This material was used because of its high mechanical strength and resistance to pressure. At that time, steel did not replace cast iron in the distribution network but rather supplemented it in key elements of the system. From the beginning of the 20th century, steel became the standard construction material for modern water supply facilities.

**Figure 1:**
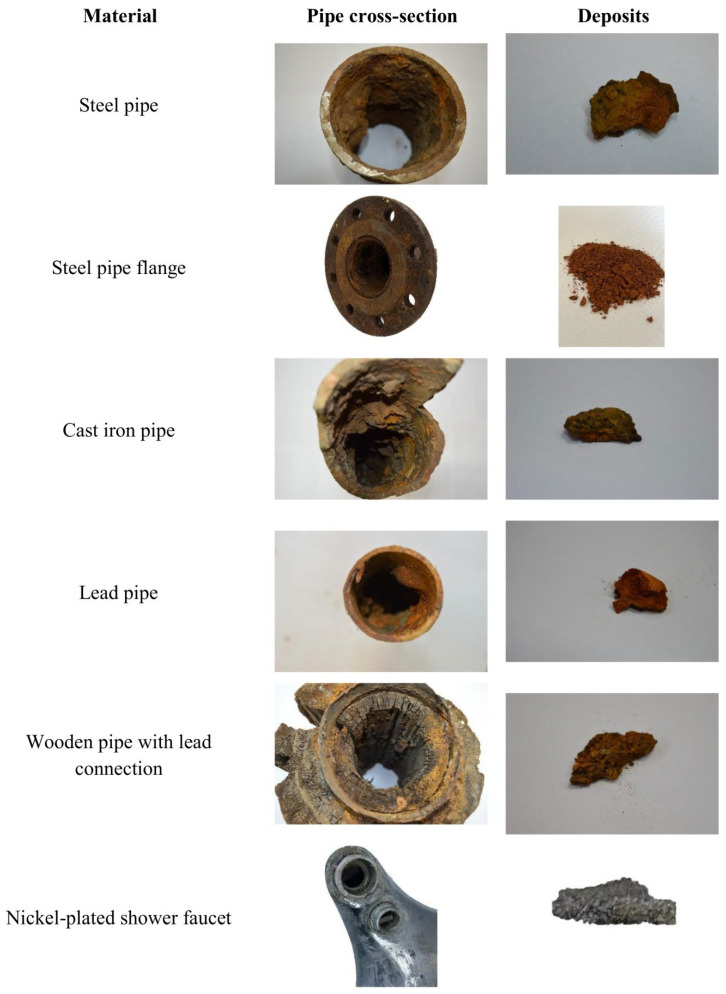
Analysed cross-sections of pipes with deposits subjected to ATR-FTIR and ICP-OES analysis.

In addition, scale deposits were also collected from infrastructure outside Świdnica. Additional samples included deposits from a steel pipe flange in Borucin (Silesian Voivodeship, Poland) and from a nickel-plated steel shower head in Racibórz (Silesian Voivodeship, Poland). A total of six corrosion scale samples were collected from various pipes, as summarised in Table 1. All pipes were used to transport water from underground intakes. Figure 1 shows the pipe sections and the corrosion scales on the pipe surfaces. All scale samples were collected from the inlet of each pipe.

### 2.2. ATR-FTIR and ICP-OES Analysis

The deposit samples were analysed in the Chemical Laboratory of Wrocław University of Environmental and Life Sciences, where ATR-FTIR analyses were performed using the Nicolet iN10 integrated infrared microscope with the external Nicolet iZ10 FT-IR module (Thermo Fischer Scientific, Waltham, MA, USA). Each spectrum represented the average of 32 scans in the 400–4000 cm^−1^ wavenumber range at a spectral resolution of 4 cm^−1^. The deposits were gently crushed to obtain a powdered material and directly placed onto the diamond ATR crystal for analyses. The determination of metal concentrations in the deposit samples was carried out using the ICP-OES method with the Thermo Scientific iCAP 7000 Series Spectrometer (Thermo Fischer Scientific (Bremen) GmbH, Bremen, Germany). For the ICP tests, 0.5 g of each deposit sample (0.25 g in the case of the wooden pipe) was used, but the concentrations of all elements were expressed as mg/g of deposit. Deposit samples were air-dried, homogenised, and subjected to acid digestion prior to analysis. For each sample, 0.5 g of deposit (0.25 g for deposit from the wooden pipe) was digested with 5 mL of concentrated nitric acid (65%, analytical grade), and the resulting solution was diluted to 50 mL with deionized water. Multi-element calibration was carried out with ICP multi-element standard solution IV (Merck KGaA, Darmstadt, Germany, lot HC67834755), and analytical quality was checked using the certified reference material TMDA-70.3 (Environmental and Climate Change, Gatineau, QC, Canada, lot 0523). The lowest calibration standard was 0.1 mg/L. Concentrations reported below this level should be treated as approximate values. Considering the sample mass and a final solution volume of 50 mL, this corresponds to approximately 0.02 mg/g for samples with a mass of 0.25 g (scale deposits from wooden pipe) and 0.01 mg/g for samples with a mass of 0.5 g.

## 3. Results

The elemental compositions of the scale samples determined by ICP are shown in Table 2. At the study design stage, no comparative elemental analysis of individual samples was planned. The ICP analysis was mainly aimed at determining the overall elemental composition of the scale deposits. Differences between samples collected from pipes with IDs 2, 3, and 5 were assessed using the non-destructive FTIR method. Therefore, the ICP results were used for interpretative purposes only, without assigning elemental compositions to the individual scale deposits collected from pipes with IDs 2, 3 and 5.

Although the concentrations reported in this study refer to solid deposits (mg/g), the release of even small amounts of pipe scale into the water phase could locally increase metal concentrations. Lead exposure is associated with neurological and developmental effects, while chromium and nickel may cause toxic effects in the liver and kidneys. Copper and zinc are essential trace elements but may cause gastrointestinal disturbances at high concentrations. According to WHO guidelines, the recommended limits in drinking water are 0.01 mg Pb/L, 0.07 mg Ni/L, 0.05 mg Cr/L, 2 mg Cu/L. Concentration of Zn between 3 and 5 mg/L is good for healthy living [27,29]. A simple estimation indicates that for example, the release of 10 mg of deposit containing 125 mg Zn/g into 1 L of water could theoretically result in a concentration of approximately 1.25 mg Zn/L, assuming complete dissolution. This illustrates that pipe deposits may act as a potential secondary source of metals in drinking water systems. However, in real distribution systems only a fraction of metals present in deposits is typically released into the water phase.

**Figure 2:**
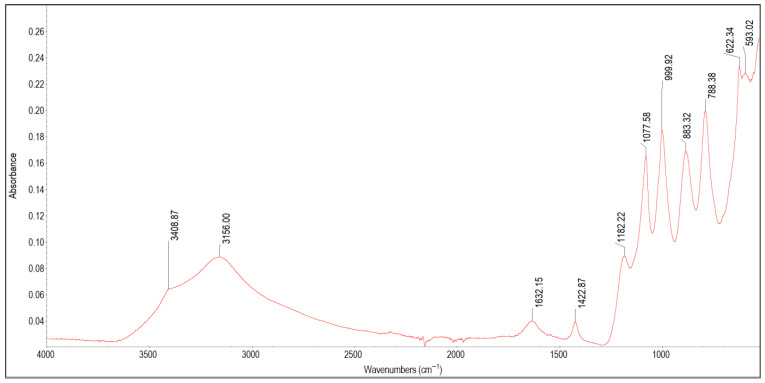
FTIR spectrum of the 1A scale deposit.

**Figure 3:**
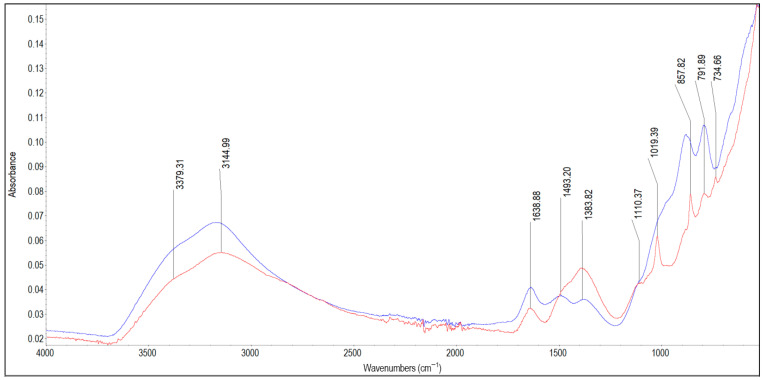
FTIR spectra of scale deposits 2A (blue) and 2B (red).

**Figure 4:**
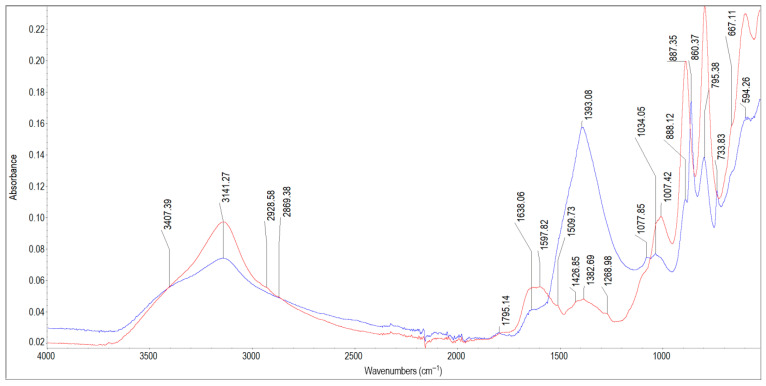
FTIR spectra of scale deposits 3A (blue) and 3B (red).

**Figure 5:**
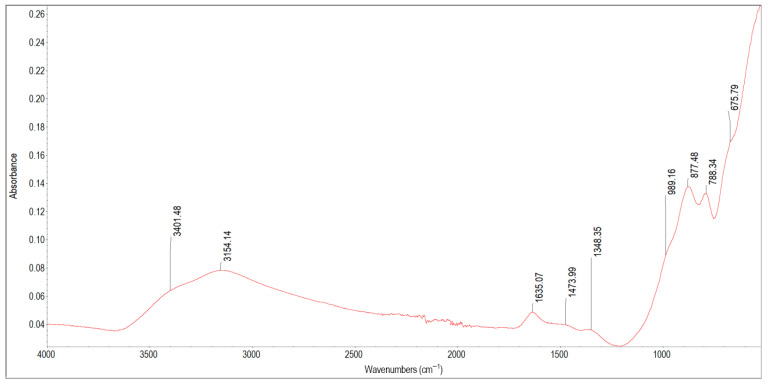
FTIR spectrum of scale deposit 4A.

**Figure 6:**
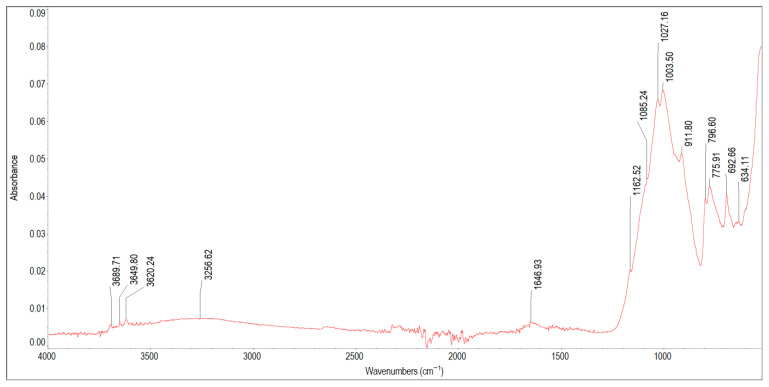
FTIR spectrum of scale deposit 5A.

**Figure 7:**
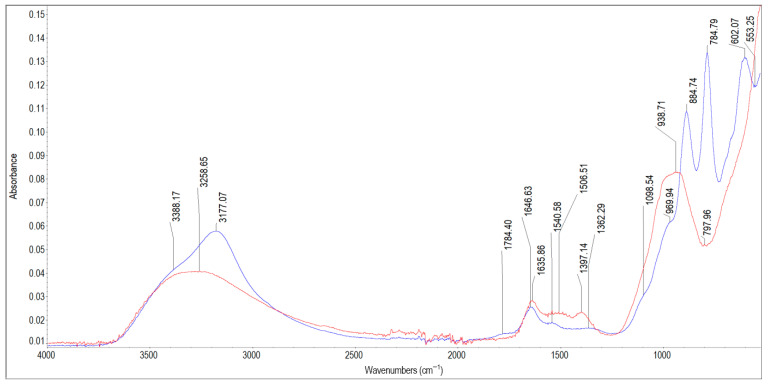
FTIR spectra of scale deposits 5B (blue) and 5C (red).

**Figure 8:**
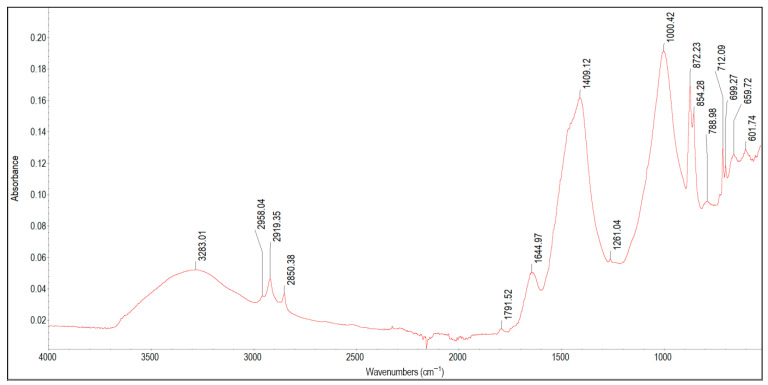
FTIR spectrum of scale deposit 6A.

The results of the FTIR analysis are presented in Figure 2, Figure 3, Figure 4, Figure 5, Figure 6, Figure 7 and Figure 8. The characteristic infrared absorption bands of the functional groups detected in the spectra are summarised in Table 3, Table 4, Table 5, Table 6, Table 7 and Table 8.

In the ATR-FTIR spectrum of sample 1A (Figure 2), the wide, triangular band with a peak at 3156 cm^−1^ and a shoulder at 3408 cm^−1^ indicates the presence of O–H hydroxyl groups or adsorbed water. Considering the very high iron content (185 mg/g), these functional groups are attributed to iron oxyhydroxides—lepidocrocite (γ-FeOOH) and goethite (α-FeOOH)—rather than to organic compounds such as polysaccharides or alcohols. The peak at 1632 cm^−1^ most likely indicates the presence of hydrous iron oxides; however, together with the peak at 1422 cm^−1^, it can be assigned to carboxylates and carboxylic acids. At lower wavenumbers, overlapping absorption bands of sulphates and phosphates (1182 cm^−1^, 1077 cm^−1^) occurred together with Si–O vibrations in silicates (1077 cm^−1^, 999 cm^−1^, 788 cm^−1^) and Fe–O vibrations in iron oxyhydroxides (883 cm^−1^ and 788 cm^−1^). The peaks at 622 cm^−1^ and 593 cm^−1^ correspond to characteristic Fe–O vibrations in iron oxyhydroxides (lepidocrocite, goethite) and oxides (maghemite, magnetite). Iron sulphates and carbonates may also be present in sample 1A. The peaks at 1422 cm^−1^ and 883 cm^−1^ may indicate the presence of small amounts of iron carbonates, as the Mg and Ca contents are negligible (0.32 mg/g and 1.5 mg/g, respectively). However, the absence of a band around 720 cm^−1^ and the dominance of silicate and oxide signals prevent the unambiguous identification of carbonates. Similarly, intense silicate and iron oxide bands of could have caused the overlap and distortion of the 622 cm^−1^ band, which—together with signals at 3156 cm^−1^, 1632 cm^−1^, 1182 cm^−1^, 1082 cm^−1^ and 1000 cm^−1^—is characteristic of jarosite, a hydrated potassium and iron sulphate [37]. Analysis of the elemental composition data shows that the concentrations of most elements (except iron) were insubstantial or present only at trace levels. Consequently, FTIR could not easily detect elements such as boron or aluminium within a mixture dominated by FeOOH and silicates, which prevailed in sample 1A. Based on FTIR and ICP analyses, sample 1A is composed mainly of goethite and lepidocrocite, together with silica and aluminosilicates, adsorbed water, possible traces of organic matter (carboxylates) and iron carbonates and sulphates.

Figure 3 depicts the spectra of scale deposits 2A and 2B collected from a steel pipe flange. The characteristic infrared absorption bands of the functional groups detected in the spectra of samples 2A and 2B are summarised in Table 4. For both samples, a broad absorption band with a peak of 3144 cm^−1^ and a shoulder at 3379 cm^−1^, together with the band at 1638 cm^−1^, indicates the presence of O–H hydroxyl groups and adsorbed water in iron oxyhydroxides, which is consistent with the ICP results (Fe = 320 mg/g). The absorption bands for sample 2A are observed at: 1493 cm^−1^, 1383 cm^−1^, 1110 cm^−1^ (shoulder), 1019 cm^−1^ (shoulder), 879 cm^−1^ and 791 cm^−1^. The bands at 1493 cm^−1^, 1383 cm^−1^ and 879 cm^−1^ have been attributed to O–H bending modes in FeOOH, but may also indicate the presence of carbonates. However, the absence of an absorption band around 720 cm^−1^, together with weaker signals at around 1493 cm^−1^ and 879 cm^−1^ resulting from band overlap, hinders unequivocal confirmation of carbonates presence in the 2A sample.

**Table 3:** Band assignment in the 1A scale deposit.

Peak Position [cm^−1^]	Literature Band Value [cm^−1^]	References	Band Assignment
3408, 3156	3400, 3142	[38]	O–H stretching vibration band of iron oxyhydroxides in lepidocrocite and goethite
1632	1632 1636 1638	[31,38,39]	O–H from adsorbed water in hydrous iron oxides C=O groups characteristic for carboxylic acids; C=C in alkenes; asymmetric stretching vibration of SO_4_^2−^
1422	1419	[40]	Vibration of C=O group of carboxylates and carboxylic acids
1422, 883	1480–1300, 881	[41,42]	Asymmetric vibration of CO_3_^2−^
1182	1180 1185	[39,43]	Symmetric stretching vibration of SO_4_^2−^ Asymmetric stretching vibration of P–O in PO_4_^3−^
1077	1075 1076 1073	[39,44,45]	Asymmetric stretching vibration of PO_4_^3−^ Asymmetric stretching vibration of P–O in HPO_4_^2−^ Symmetric stretching vibration of SO_4_^2−^
1077, 788	~1087, 800	[46]	Asymmetric and symmetric stretching of Si–O–Si
999	1006, 1001	[47,48]	Si–O in silicates, Si/Al–O and aluminosilicates
788	795	[47]	Si–C in silicates
883, 788	892, 795	[38,49]	Fe–O–H bending vibrations in α-FeOOH
622, 593	616–635, 580, 592	[38,50]	Characteristic vibration band of Fe-O in lepidocrocite, goethite, maghemite

The shoulders at 1110 cm^−1^ and 1019 cm^−1^ are characteristic of clay minerals; however, their shapes indicate poor crystallinity, as the peaks are not sharp. Sharper peaks at 879 cm^−1^ and 791 cm^−1^ reflect the presence of more crystalline goethite. The spectrum of sample 2B shows less distinct peaks at 857 cm^−1^ and 791 cm^−1^ and more pronounced peaks at 1110 cm^−1^ and 1019 cm^−1^, which no longer appear as shoulders. A broader and more intense band at 1383 cm^−1^ in the spectrum of sample 2B corresponds to more disordered FeOOH. Taken together, these observations indicate that sample 2A contains a more crystalline form of FeOOH (most likely goethite) and lower proportion of silica (weaker peaks at 1110 cm^−1^ and 1019 cm^−1^). In contrast, sample 2B contains more disordered FeOOH (broad 1383 cm^−1^), more silicates (stronger peaks at 1110 cm^−1^ and 1019 cm^−1^) and less goethite. Under aerobic conditions, iron compounds become more crystalline, whereas under reducing conditions amorphous FeOOH structures are formed [51,52]. Consequently, the layer of deposit closer to the water interface is more crystalline. Silica decreases the rate of Fe^2+^ oxidation to Fe^3+^ and affects both the crystallinity and size of iron oxides [53]. It also exhibits a higher sorption capacity towards poorly crystalline FeOOH, slowing down its transformation into more crystalline forms, as demonstrated by Cornell et al. (1987), who investigated the effect of silicate species on the transformation of ferrihydrite into goethite [54]. The higher sorption capacity of silica on ferrihydrite compared to goethite results from its much larger specific surface area (up to 700 m^2^/g versus up to 200 m^2^/g for goethite) [55]. On this basis, it can be stated that sample 2B was located closer to the pipe wall than sample 2A, which probably did not have direct contact with water, as no crystalline lepidocrocite was detected.

**Table 4:** Band assignment in scale deposits 2A and 2B.

Peak Position [cm^−1^]	Literature Band Value [cm^−1^]	References	Band Assignment
3379, 3144	3400, 3142	[38]	O–H stretching vibration band of iron oxyhydroxides in lepidocrocite and goethite
1638	1632	[38]	O–H from adsorbed water
1493, 1383	1482, 1389	[42]	asymmetric stretching vibration of carbonates
1493, 1383	~1500–1400	[38,50]	O–H bending vibration in FeOOH lattice structure
shoulders 1110, 1019	1120–1000	[56]	Si–O stretching vibrations in clay minerals
1019	1021, 1018	[38,50]	O–H bending vibration in lepidocrocite
879, 791	892, 795	[38,49]	Fe–O–H bending vibrations in α-FeOOH lattice structure
879, 857, 734	881, 853, 730	[41]	Asymmetric deformation of CO_3_^2−^
734	740, 733	[50,57]	bending vibration of O–H in lepidocrocite

Figure 4 shows the spectra of scale deposits 3A and 3B collected from a wooden pipe. The characteristic infrared absorption bands of the functional groups detected in the spectra of samples 3A and 3B are summarised in Table 5. For both samples, a broad absorption band with a peak of 3141 cm^−1^ and a shoulder at 3407 cm^−1^ indicates the presence of the O–H hydroxyl groups and adsorbed water in iron oxyhydroxides, which is consistent with the ICP results (Fe = 125 mg/g). The absorption bands for sample 3A are observed at: 1795 cm^−1^, 1638 cm^−1^, 1393 cm^−1^, 1077 cm^−1^, 1034 cm^−1^, 888 cm^−1^ (shoulder), 860 cm^−1^, 795 cm^−1^, 733 cm^−1^, 667 cm^−1^ (shoulder), and 594 cm^−1^. The peak at 1795 cm^−1^ can be assigned to a combination overtone in iron oxyhydroxides. The peak at 1638 cm^−1^ can be attributed to weakly bound water in hydrous iron oxides; however, together with the peak at 1034 cm^−1^, it indicates the presence of organic matter (lignin and cellulose from wood). The bands found at 1393 cm^−1^, 888 cm^−1^, 860 cm^−1^, 733 cm^−1^ and 667 cm^−1^ most likely correspond to iron and calcium carbonates. The peak at 667 cm^−1^ also indicates the mixture of iron oxides and carbonates. The peaks at 1077 cm^−1^, 1034 cm^−1^ and 795 cm^−1^ testify to the presence of silicates, with partial overlap from Fe–O vibrations in iron oxides and oxyhydroxides (peaks at 795 cm^−1^, 594 cm^−1^). Small, broad peaks at 1077 cm^−1^ and 1034 cm^−1^ are characteristic of amorphous silica [56]. The FTIR spectrum of sample 3B shows bands at 2929 cm^−1^ and 2869 cm^−1^, 1795 cm^−1^, ~1600 cm^−1^, 1426 cm^−1^, 1382 cm^−1^, 1268 cm^−1^, 1007 cm^−1^, 887 cm^−1^, 795 cm^−1^, 667 cm^−1^ (shoulder) and 594 cm^−1^. The absorption bands at 2929 cm^−1^, 2869 cm^−1^, 1795 cm^−1^, 1600 cm^−1^, 1426 cm^−1^ and 1382 cm^−1^ can be attributed to organic matter, most likely biofilm residues, bacterial extracellular polymeric substances (EPS), humic substances (aromatic C=C at 1600 cm^−1^, C–H stretching at 2929 cm^−1^ and 2869 cm^−1^) as well as lignin- and cellulose-related compounds originating from the wooden material. The bands at 2929 cm^−1^ and 2869 cm^−1^ correspond to aliphatic C–H stretching vibrations commonly present in many types of organic matter. The band at 1007 cm^−1^ indicates the presence of crystalline silica or aluminosilicates. Strong and sharp peaks at 887 cm^−1^ and 795 cm^−1^ are fingerprints of well-crystallised goethite. The peaks at 3147 cm^−1^ and 594 cm^−1^ in sample 3B are slightly higher and sharper than those in sample 3A, which indicates a higher degree of crystallinity of iron compounds. Considering the higher crystallinity of iron oxyhydroxides and silica, as well as the presence of bound organic matter from the biofilm and the near absence of carbonates, it can be concluded that sample 3B originated from an outer layer, closer to the water interface, whereas sample 3A was located closer to the wooden pipe wall.

Figure 5 presents the spectrum of scale deposit 4A collected from a lead pipe. The characteristic infrared absorption bands of the functional groups detected in the spectrum of sample 4A are summarised in Table 6. The weak signals observed in the spectrum indicate that the deposit layer was thin and, despite contact with the pipe wall, was also exposed to flowing water. This explains the broad and low bands, which suggest strong hydration, low crystallinity, a high content of amorphous components, and a low content of carbonates. As in the previously discussed samples, sample 4A also contains hydrated FeOOH (peaks at 3401 cm^−1^, 3154 cm^−1^, 1635 cm^−1^, 1473 cm^−1^, 1348 cm^−1^, 877 cm^−1^, 788 cm^−1^, 675 cm^−1^), which occurs mainly as ferrihydrite and poorly crystalline goethite. The peak at 1348 cm^−1^ is attributed to Si–O–Fe vibrations resulting from the adsorption of silica by FeOOH. The absorption bands at 1473 cm^−1^ and 1348 cm^−1^ can also indicate the presence of organic matter (lipids and proteins) from biofilm, as well as carbonates. The peaks at 788 cm^−1^ and 675 cm^−1^ can also be assigned to carbonates, primarily iron and calcium carbonates, as these two elements dominate the deposit (218.5 mg/g and 3.76 mg/g, respectively). Reliance on FTIR alone could have led to a misleading mineralogical interpretation.

**Table 5:** Band assignment in scale deposits 3A and 3B.

Peak Position [cm^−1^]	Literature Band Value [cm^−1^]	References	Band Assignment
3407, 3141	3400, 3142	[38]	O–H stretching vibration band of oxyhydroxides in lepidocrocite and goethite
2928, 2869	3000–2800	[31,58]	aliphatic or alicyclic C–H stretching in saccharides, polyalcohols and fats; stretching of aliphatic C–H bond in humic acid
1795	1799 ~1790	[38,59]	Asymmetric vibration of organic C=O Combination band in goethite
1638	1636 1634 1632	[31,38,58]	O–H bending vibration of water adsorbed by cellulose C=O groups characteristic for carboxylic acids; C=C in alkenes; O–H from adsorbed water in hydrous iron oxides
1597	1577 1575	[31,60]	Fe–O–H bending vibration in ferrihydrite COO^−^ in carboxylic groups
1426, 1382	1437, 1383	[58]	C–H vibration in aromatic skeleton of cellulose
1393	1389	[42]	asymmetric stretching vibration of carbonates
1268	1241	[58]	C–O stretching of guaiacyl ring in lignin
1077	~1087	[46]	Asymmetric and symmetric stretching of Si–O–Si
1034	1030	[31,58]	Si–O stretching in the mineral phase of the sludge (silicate impurities and clay minerals possibly in a complex with humic acids); C–O stretching of polysaccharides or polysaccharide-like substances (e.g., lignin, cellulose)
1007	1006, 1001	[47,48]	Si–O in silicates, Si/Al–O and aluminosilicates
784	795	[47]	Si–C in silicates
887, 795	892, 795	[38,49]	Fe–O–H bending vibrations in α–FeOOH
888, 860, 733	881, 853, 730	[41]	Asymmetric deformation of CO_3_^2−^
667	670 680	[41,49]	α-Fe_2_O_3_ Symmetric deformation of CO_3_^2−^
594	592 580	[38,50]	vibration band of Fe–O in magnetite vibration band of Fe–O in maghemite

The bands at 3154 cm^−1^, 1635 cm^−1^, 1473 cm^−1^, 1348 cm^−1^, 877 cm^−1^ and 788 cm^−1^ form a pattern that is highly characteristic of hydrated lead carbonate corrosion products [42,61]. Without additional compositional data, spectral identification could point to the presence of hydrocerussite or Pb–CO_3_ compounds. However, ICP-OES analysis showed that lead was present only in trace amounts (0.003 mg/g), and therefore the absorption bands in the FTIR spectrum are attributed not to lead carbonates but to overlapping absorptions from iron oxyhydroxides, minor amounts of carbonates and organics from biofilm. This shows that FTIR alone is insufficient to distinguish between compounds with different chemical composition but similar infrared responses. Accordingly, it is necessary to combine infrared spectroscopy with elemental analysis when studying corrosion scale deposits in water pipes.

**Table 6:** Band assignment in scale deposit 4A.

Peak Position [cm^−1^]	Literature Band Value [cm^−1^]	References	Band Assignment
3401, 3154	3400, 3142	[38]	O–H stretching vibration band of iron oxyhydroxides in lepidocrocite and goethite
1635	1632 1636	[31,38]	O–H from adsorbed water in hydrous iron oxides C=O groups characteristic for carboxylic acids; C=C in alkenes;
1473, 1348	1453, 1383	[38]	Fe–O–H bending vibrations in goethite
1473, 1348	1480–1350 1455, 1398	[62,63]	Alkane C–H bending vibrations of proteins, lipids
1348	1347	[64]	Fe–O as Si cage(Si–O–Fe) in ferrisilicates
877, 788	892, 795	[38,49]	Fe–O–H bending vibrations in α-FeOOH
675	670 680 679	[41,49]	α-Fe_2_O_3_ Symmetric deformation of CO_3_^2−^

Figure 6 and Figure 7 depict the spectra of scale deposits 5A, 5B, and 5C collected from a cast iron pipe. The characteristic infrared absorption bands of the functional groups detected in the spectra of samples 5A, 5B and 5C are listed in Table 7. The FTIR spectrum of sample 5A shows essentially no characteristic peaks attributable to iron compounds, even though the average iron content in all cast iron deposits was 147.56 mg/g. Only a small peak at 634 cm^−1^ corresponds to Fe–O vibrations in this sample. The remaining bands originate chiefly from silica (Si–O vibrations at 1162 cm^−1^, 1085 cm^−1^, 775 cm^−1^, 692 cm^−1^) and from kaolinite (inner surface and inner hydroxyls at 3689 cm^−1^, 3649 cm^−1^, 3620 cm^−1^; adsorbed water at 3256 cm^−1^ and 1646 cm^−1^; Si–O stretching at 1027 cm^−1^, 1003 cm^−1^; vibration of Al_2_OH at 911 cm^−1^). In the spectrum of sample 5B, characteristic peaks of iron oxyhydroxides can be observed: 3177 cm^−1^ with a shoulder at 3388 cm^−1^ and 1646 cm^−1^ (O–H hydroxyl groups and adsorbed water), and peaks at 884 cm^−1^ and 784 cm^−1^ (characteristic goethite spectrum doublet). The 1362 cm^−1^ band reflects Si–O–Fe vibrations in ferrisilicates, while the band at 602 cm^−1^ is attributed to Fe–O vibrations. The shoulder at 1098 cm^−1^ can be assigned to Si–O vibrations in amorphous silica. The bands at 1646 cm^−1^ and 1540 cm^−1^ may also indicate the presence of organic matter (proteins) in sample 5B, most likely originating from a biofilm. This interpretation is supported by a very weak absorption band near 2930 cm^−1^, corresponding to C–H stretching vibrations in organics. In mineral-rich deposits, such as sample 5B, amide bands (1646 cm^−1^ and 1540 cm^−1^) appear as broad and flat peaks, while the C–H stretching band around 2930 cm^−1^ may be strongly suppressed or almost completely absent [65]. Sample 5C contains poorly crystalline iron oxyhydroxides, as evidenced by a broad and small peak around 3258 cm^−1^, peaks at 1506 cm^−1^ and 1397 cm^−1^ reflecting the O–H bending vibration in the FeOOH lattice, and a broad, flat-topped peak characteristic of ferrihydrite around 938 cm^−1^. The peak at 1635 cm^−1^ is most likely associated with O–H vibrations of adsorbed water in hydrous iron oxides; however, together with the peak at 938 cm^−1^, it can indicate the presence of clay minerals. Sample 5C may also contain small amounts of carbonates (peaks at 1506 cm^−1^, 1397 cm^−1^). Taking into account the three scale deposits collected from the inner surface of the cast iron pipe wall, it can be concluded that sample 5C was located closest to the pipe wall, as it contains the highest amount of poorly crystalline FeOOH (most likely ferrihydrite). Above it lies sample 5B, characterised by well-crystalline goethite (peaks 884 cm^−1^, 784 cm^−1^) and more crystalline iron oxyhydroxides (sharper peak ca. 3177 cm^−1^). The layer closest to the water interface is sample 5A, which contains no iron compounds from pipe corrosion but consists predominantly of silica and kaolinite. This layer reduced iron release from the pipe and acted similarly to a protective film on pipe surfaces [53].

**Table 7:** Band assignment in scale deposits 5A, 5B and 5C.

Peak Position [cm^−1^]	Literature Band Value [cm^−1^]	References	Band Assignment
3689, 3649, 3620	3695, 3653, 3620	[56]	Stretching vibrations of inner surface O–H bond in clay minerals
3388, 3258, 3177	3400, 3142, 3500–3100	[38,56]	O–H stretching vibration band of iron oxyhydroxides in lepidocrocite and goethite Stretching vibrations of H-bonded water
1784	~1790	[38]	Combination band in goethite
1635, 1646	1632 1638	[38,56]	O–H from adsorbed water H–O–H bending vibrations of water molecules adsorbed on clay minerals
1646, 1540	1650–1590, 1560–1500	[62]	amide group (O=CH–NH), peptide bond vibrations
1506, 1397	~1500–1400	[38,50]	O–H bending vibration in FeOOH lattice structure
1506, 1397, 1362	1482, 1389, 1365	[42]	asymmetric stretching vibration of carbonates
1362	1347	[64]	Fe–O as Si cage(Si–O–Fe) in ferrisilicates
1162	1155	[66]	asymmetric stretching vibration of Si–O–Si
1098, 1085	1083	[56]	Si–O vibrations of amorphous silica
1027	1021	[57]	bending vibration of O–H in lepidocrocite
1027, 1003	1033, 1011	[56]	Si–O stretching vibrations of kaolinite
938	936 934, 930	[56,67]	Al_2_OH bending band of dickite Characteristic peak of ferrihydrite
911	916	[56]	bending vibration of Al_2_OH in kaolinite
884, 784	892, 795	[38,49]	Fe–O–H bending vibrations in α-FeOOH
784	784	[47]	Si-C in silicates
775, 692	758 693	[66]	symmetric stretching vibration of Si–O–Si bending vibration of Si–O–Si
634	635 630	[38,49]	Fe–O stretching vibrations in α-FeOOH (goethite)
602	592	[38]	vibration band of Fe–O in magnetite

Figure 8 presents the spectrum of scale deposit 6A collected from a nickel-plated shower faucet. The characteristic infrared absorption bands of the functional groups detected in the spectrum of this sample are presented in Table 8.

**Table 8:** Band assignment in scale deposit 6A.

Peak Position [cm^−1^]	Literature Band Value [cm^−1^]	References	Band Assignment
3283	3600–3200 3350, 3400, 3409	[31,68,69,70]	O–H group in polymeric compounds (polysaccharides, phenols, polyalcohols) and in adsorbed water O–H groups of adsorbed water in magnesium, calcium and zinc hydroxides
2958, 2919, 2850	~2915, ~2850	[71,72,73]	Stretching vibrations of C–H in zinc, magnesium and other metal-stearates
1791	1799	[74]	Combination bands of carbonate species
1644	~1640	[31,56,75]	C=O groups characteristic for carboxylic acids; C=C in alkenes H–O–H bending vibrations of water molecules adsorbed on clay minerals O–H bending vibrations in metal hydroxides
1409	1405–1415	[41,76,77]	Asymmetric stretching vibration of carbonates
1000,	1006, 1001	[47,48]	Si–O in silicates, Si/Al–O and aluminosilicates
788	795 ~800	[47,56]	Si–C in silicates Amorphous silica in clay minerals
872, 854	881, 853	[41]	Asymmetric deformation of CO_3_^2−^
712, 699, 659	716, 696, 668	[73]	bending vibrations of COO^−^ and rocking vibration of CH_2_ in metal-stearate salts
601	608	[78]	Stretching vibrations of Zn–O

In the spectrum of sample 6A, the following bands can be observed: 3283 cm^−1^, 2958 cm^−1^, 2919 cm^−1^, 2850 cm^−1^, 1791 cm^−1^, 1644 cm^−1^, 1409 cm^−1^, 1000 cm^−1^, 872 cm^−1^, 854 cm^−1^, 788 cm^−1^, 712 cm^−1^, 699 cm^−1^, 659 cm^−1^, and 601 cm^−1^. The broad band with a peak at 3283 cm^−1^ can be assigned to O–H hydroxyl groups and adsorbed water in polymeric compounds, as well as to Ca, Mg and Zn hydroxides, which is consistent with the ICP results showing high concentrations of Zn, Ca and Mg and negligible content of Fe (Zn = 124.58 mg/g, Ca = 76.34 mg/g, Mg = 3.24, Fe = 0.083 mg/g). The peaks at 2958 cm^−1^, 2929 cm^−1^ and 2869 cm^−1^ are attributed to aliphatic C–H stretching vibrations typical of organic compounds. In this case, considering the sharp character of these bands and the literature reports, they are most likely associated with Ca, Mg, and Zn stearates or related fatty residues. The peaks at 712 cm^−1^, 699 cm^−1^ and 659 cm^−1^ also indicate the presence of metal-stearate salts, i.e., soap residues. Sample 6A also contains silicates or aluminosilicates (peaks at 1000 cm^−1^, 788 cm^−1^). Ca, Mg, and Zn carbonates can be identified at 1791 cm^−1^, 1409 cm^−1^, 872 cm^−1^ and 854 cm^−1^. The peak at 601 cm^−1^ corresponds to Zn–O bonds. Based on the combined FTIR and ICP analyses, sample 6A is chiefly a mixture of soap-derived organic residues, silica and aluminosilicates, adsorbed water, and Ca, Mg, and Zn carbonates, with no evidence for significant Fe-containing minerals.

The presence of organic matter such as proteins, lipids and polysaccharides detected in the FTIR spectra of several scale deposits is most likely associated with biofilm components, including EPS. Although these compounds themselves are not typically considered harmful, biofilms in drinking water systems may provide a favourable environment for microbial growth and the persistence of opportunistic pathogens. Moreover, the organic matter may participate in reactions with disinfectants (potentially contributing to the formation of carcinogenic disinfection by-products) and may contribute to the formation of taste- and odour-causing substances. Therefore, the occurrence of biofilm-derived organic matter in pipe deposits may indirectly influence drinking water quality [14].

The detected functional groups and mineral phase can be related to the corrosion behaviour of the pipe materials and the subsequent evolution of corrosion scales. Iron oxyhydroxides identified in the spectra represent typical corrosion products of iron-based materials, whereas silica-rich layers and organic functional groups indicate secondary deposition processes and the presence of biofilm-derived organic matter. These processes contribute to the stratified structure of the deposits observed in the studied pipe systems.

## 4. Conclusions

The combined application of FTIR and ICP-OES proved useful for characterising the composition of scale deposits in water pipes.

FTIR enabled differentiation between various mineral phases in multilayered scale deposits, including amorphous and poorly crystallised deposits such as ferrihydrite, and well-crystallised goethite and lepidocrocite. ICP analysis confirmed that Fe, Ca, Mg, Zn and Al were the principal elements that formed these phases. This allowed reconstruction of how the deposits were layered within the pipes. Poorly crystalline FeOOH, together with amorphous silica, carbonates, phosphates, and sulphates, dominated the layers closest to the pipe walls, whereas more crystalline forms of FeOOH, such as goethite and lepidocrocite, appeared nearer the water interface. In this respect, FTIR proved to be particularly effective for determining inner and outer layers of deposits, even when samples had been collected without knowledge of their original location.

However, FTIR alone could lead to incorrect interpretations of compounds that differ in chemical composition but overlap in their infrared responses, as exemplified by the analysis of scale deposit from a lead pipe. Without information on elemental composition, spectral analysis could have led to the misidentification of lead carbonates, whereas the lead content was negligible and practically undetectable by FTIR. In the lead pipe deposit sample, the FTIR signals were primarily attributed to FeOOH, carbonates and biofilm residues. Conversely, elemental analysis alone does not provide information on chemical compounds and their structure. The application of FTIR made it possible to identify organic matter originating as residues from organic processes such as those accumulating in biofilms.

Scale deposits from the wooden pipe were a mixture of organic and mineral compounds. The presence of cellulose- and lignin- related bands with iron oxyhydroxides and iron/calcium carbonates was detected in these samples. To the authors’ knowledge, this study presents the first reported FTIR and ICP characterisation of scale deposits from a wooden water pipe.

Silica was found to have a strong influence on the crystallinity and phase transformations of FeOOH. Samples with higher silica content exhibited more disordered FeOOH, most likely because silica slowed down its transformation into more crystalline forms, suggesting that these layers were located closer to the pipe wall. However, sample 5A from the cast iron pipe, composed almost entirely of well-crystallised silica and kaolinite with negligible iron content, formed a surface crust that limited the release of iron corrosion products from underlying layers, indicating its location near the water interface.

ICP analysis enabled the detection of harmful elements in the deposits; however, these were present in trace amounts, meaning that their leaching would be unlikely to pose significant health risks under normal conditions Nevertheless, the potential mobilisation of pipe deposits can locally increase metal concentrations in the water phase, indicating that such deposits can act as a secondary source of metals in drinking water systems. Therefore, pipe scale deposits have the potential to indirectly influence drinking water quality and human exposure to trace metals.

The combined use of FTIR and ICP-OES proved to be a powerful tool for analysing corrosion scales. It enabled the identification of mineral and organic constituents and the reconstruction of deposit stratification in both historical and modern water distribution systems.

Improved understanding of the processes governing corrosion scale formation, composition, and stratification is important, as these factors influence pipe hydraulics and corrosion rates. Consequently, the combined application of FTIR and ICP-OES can support pipe maintenance strategies, including water supply network cleaning, corrosion mitigation, and biofilm control.

## Figures and Tables

**Table 1:** Characteristics of the analysed pipes.

Pipe ID	Sample ID	Material	Internal Pipe Diameter [mm]	Pipe Age
1	A	Steel pipe	150	~60 years
2	A B	Steel pipe flange	100	the 1980s
3	A B	Wooden pipe with lead connection	100	before 1864
4	A	Lead pipe	50	after the year 1800
5	A B C	Cast iron pipe	125	~100 years
6	A	Nickel-plated shower faucet	-	20 years

**Table 2:** Elemental compositions of scale samples determined by ICP test (mg/g of deposit).

Element	Steel Pipe	Steel Pipe Flange	Wooden Pipe with a Lead Inlet	Lead Pipe	Cast Iron Pipe	Nickel- Plated Shower Faucet
Ca	1.5	6.62	17.36	3.76	2.04	76.338
Mg	0.32	0.416	1.236	0.418	0.36	3.24
Fe	185.84	320.62	125.08	218.54	147.56	0.083
Cu	<0.01	0.077	<0.02 *	<0.01 *	0.008	0.933
Ni	<0.01 *	0.035	<0.02 *	<0.01 *	<0.01 *	1.259
Cr	0.011	0.015	<0.02 *	0.014	0.009	<0.01 *
Mn	0.169	0.475	0.349	0.074	1.6	0.004
Al	0.114	0.174	0.339	0.139	0.188	0.944
Zn	0.003	0.41	0.002	0.266	0.014	124.583
Pb	<0.01 *	<0.01 *	0.036	0.003	<0.01 *	0.003
Sr	<0.01 *	0.019	<0.02 *	0.002	<0.01 *	0.198
Ba	0.033	0.149	0.027	0.146	0.21	<0.01 *
Ga	0.011	0.019	0.002	0.012	0.007	<0.01 *
B	0.849	<0.01 *	0.657	1.057	0.681	<0.01 *

* Values below the practical quantification limit. For samples with a mass of 0.5 g, this limit corresponds to <0.01 mg/g, and for 0.25 g samples to <0.02 mg/g. Values reported numerically below this limit (e.g., 0.002 mg Zn/g) are approximate and were obtained by extrapolation of the calibration curve.

## Data Availability

The original contributions presented in this study are included in the article. Further inquiries can be directed to the corresponding author.

## Abbreviations

The following abbreviations are used in this manuscript:

ATR-FTIR	Attenuated Total Reflection–Fourier Transform Infrared
EDS	Energy Dispersive X-ray Spectroscopy
ICP-OES	Inductively Coupled Plasma–Optical Emission Spectrometry
SEM	Scanning Electron Microscopy
XPS	X-ray Photoelectron Spectroscopy
XRD	X-ray Diffraction
XRF	X-ray Fluorescence
